# Roles for diacylglycerol in synaptic vesicle priming and release revealed by complete reconstitution of core protein machinery

**DOI:** 10.1073/pnas.2309516120

**Published:** 2023-08-17

**Authors:** R. Venkat Kalyana Sundaram, Atrouli Chatterjee, Manindra Bera, Kirill Grushin, Aniruddha Panda, Feng Li, Jeff Coleman, Seong Lee, Sathish Ramakrishnan, Andreas M. Ernst, Kallol Gupta, James E. Rothman, Shyam S. Krishnakumar

**Affiliations:** ^a^Nanobiology Institute, Yale University School of Medicine, New Haven, CT 06520; ^b^Department of Cell Biology, Yale University School of Medicine, New Haven, CT 06520; ^c^Department of Pathology, Yale University School of Medicine, New Haven, CT 06520; ^d^School of Biological Sciences, University of California San Diego, San Diego, CA 92093; ^e^Department of Neurology, Yale University School of Medicine, New Haven, CT 06520

**Keywords:** neurotransmitter release, SNARE protein, vesicle priming, Munc13, Munc18

## Abstract

Here, we introduce the full functional reconstitution of genetically validated core protein machinery (SNAREs, Munc13, Munc18, Synaptotagmin, and Complexin) for synaptic vesicle priming and release in a geometry that enables detailed characterization of the fate of docked vesicles both before and after release is triggered with Ca^2+^. Using this setup, we identify new roles for diacylglycerol (DAG) in regulating vesicle priming and Ca^2+^-triggered release involving the SNARE assembly chaperone Munc13. We find that low concentrations of DAG profoundly accelerate the rate of Ca^2+^-dependent release, and high concentrations reduce clamping and permit extensive spontaneous release. As expected, DAG also increases the number of docked, release-ready vesicles. Dynamic single-molecule imaging of Complexin binding to release-ready vesicles directly establishes that DAG accelerates the rate of SNAREpin assembly mediated by chaperones, Munc13 and Munc18. The selective effects of physiologically validated mutations confirmed that the Munc18–Syntaxin–VAMP2 “template” complex is a functional intermediate in the production of primed, release-ready vesicles, which requires the coordinated action of Munc13 and Munc18.

## Significance

SNARE-associated chaperones, Munc13 and Munc18, act as “priming” factors, facilitating the formation of a pool of docked, release-ready vesicles and regulating Ca^2+^-evoked neurotransmitter release. Although important insights into Munc18/Munc13 function have been gained, how they assemble and operate together remains enigmatic. To address this, we developed a biochemically defined fusion assay, which enabled us to investigate the cooperative action of Munc13 and Munc18 in molecular terms. We find that Munc18 nucleates the SNARE complex, while Munc13 promotes and accelerates the SNARE assembly in a DAG-dependent manner. The concerted action of Munc13 and Munc18 stages the SNARE assembly process to ensure efficient “clamping” and formation of stably docked release-ready vesicles, which can be triggered to fuse rapidly (~10 ms) upon Ca^2+^ influx.

Information transfer in the brain depends on the controlled yet rapid (millisecond) release of neurotransmitters stored in synaptic vesicles (SVs). SV fusion is mediated by synaptic SNARE proteins, VAMP2 (v-SNARE) on the vesicles and Syntaxin/SNAP25 (t-SNAREs) on the target presynaptic membrane (1, 2). When an SV approaches the plasma membrane (PM), the helical SNARE motifs of the v- and t-SNAREs progressively assemble (“zipper”) into a ternary complex, which provides the energy to fuse the opposing membranes (3, 4). Neurotransmitter release is tightly controlled by presynaptic calcium (Ca^2+^) concentration and occurs from a readily release pool (RRP) of vesicles that are “primed” to rapidly fuse upon Ca^2+^-influx (5–8).

The “priming” process involves a series of preparatory molecular reactions that properly align the v- and t-SNAREs for polarized assembly and arrests (“clamps”) SNARE zippering in an intermediate state (9–12). This ensures that every RRP vesicle contains multiple partially assembled “SNAREpins” close to the point of triggering fusion, which can be synchronously released by Ca^2+^ to drive ultrafast fusion (13). Vesicle priming is orchestrated by molecular chaperones (Munc13 and Munc18) which guide the SNARE assembly process, and regulatory elements (Synaptotagmin and Complexin) which act together to “clamp” the SNARE assembly to produce RRP vesicles (14–17). The nucleation of the SNARE assembly has been shown to be error-prone and slow; thus, the combined action of specialized chaperones, Munc18 and Munc13 is crucial for efficient priming (16).

Munc18 forms a tight binary complex with Syntaxin, which is essential for proper Syntaxin folding and recruitment of the Munc18/Syntaxin complex to the active zone (18–20). This interaction also maintains Syntaxin in a “closed” conformation, preventing premature binding with SNAP25 on the presynaptic membrane (15, 17, 21). Recent structural analyses, which revealed that Munc18 can simultaneously bind the Syntaxin and VAMP2 SNARE motifs (22, 23), suggest that Munc18 may also serve as a template for nucleating SNARE assembly by holding Syntaxin and VAMP2 in proper alignment. The functional importance of the Munc18–Syntaxin–VAMP2 “template” complex has been inferred by single-molecule force spectroscopy and bulk-fusion assays (24–26).

Munc13, a large multidomain protein (21, 27–29), also plays a key and multifaceted role in the SV priming process. Specifically, Munc13 catalyzes the SNARE nucleation by “opening” the closed Syntaxin/Munc18 complex to enable the formation of the Munc18-Syntaxin-VAMP2 “template” complex (16, 17, 21, 30). Munc13 also promotes its progression into a “ternary” SNARE complex with SNAP25 (16). This catalytic function and associated SNARE interactions have been mapped to the large central module within Munc13 called the MUN domain (30). Additionally, Munc13 is believed to be involved in the initial tethering and docking of SV at the active zone by acting as a “molecular bridge” that links the SV and PM. This function is thought to involve interactions between the C1- C2B region and the C2C domain located on opposite ends of the MUN domain (17, 27, 31). The conserved C1-C2 domains have been shown to mediate interactions with lipids [e.g., phosphatidylserine (PS), phosphatidylinositol 4,5-bisphosphate (PIP2), diacylglycerol (DAG)], Ca^2+^, and other proteins as a means of modulating Munc13 docking/priming function (17, 32–36). Accumulating evidence shows that activation of these domains, separately or in combination, has a profound stimulatory effect on the replenishment and priming of the RRP vesicles both during and after high neuronal activity (37, 38). Recently, distinct oligomeric assemblies of Munc13 have been described, which are likely to function at distinct stages of vesicle priming and to be differentially regulated by DAG (27, 36).

The precise roles of Munc13 and its ligand DAG in vesicle priming and release remain to be established. This is largely due to the overlapping nature of their functions and a lack of functional reconstitutions with sufficient resolution to dissect their contributions at different stages of vesicle docking, priming, and fusion. We have recently described a biochemically fully defined experimental setup based on a suspended lipid membrane (SLIM) platform that is well suited for this purpose (39–42). This high-throughput, cell-free platform can precisely track individual vesicle docking, priming (clamping) and Ca^2+^-triggered release events with millisecond resolution using fluorescence microscopy. Tracking of single vesicles allows for isolated measurements of vesicle docking, clamping/priming, and Ca^2+^-triggered fusion, independent of any alteration in the preceding or subsequent steps. When combined with total internal reflection fluorescence (TIRF) microscopy, it also enables the visualization of the dynamics and organization of individual proteins beneath the docked vesicles (41). The approach is highly versatile in that all critical components, including the identity and density of protein, and the composition of buffers and membranes, can be rigorously controlled and thus, allows us to build experimentally constrained molecular models, which are tightly correlated with physiology through mutational effects. Indeed, we recently employed this in vitro setup to dissect the dual clamp/activator function of Synaptotagmin and Complexin in molecular terms and develop detailed mechanistic models (13, 42).

Here, using the current iteration of the in vitro single-vesicle fusion assay, which accurately reproduces the physiological start point for SNARE recruitment and priming, we report that DAG is an essential cofactor for Munc13 chaperone function and the balanced activities of Munc18 and Munc13 are required for efficient SNARE priming and Ca^2+^-evoked vesicular release.

## Results

### Reconstitution of Munc13-Regulated Vesicle Docking and Fusion.

**Fig. 1.**
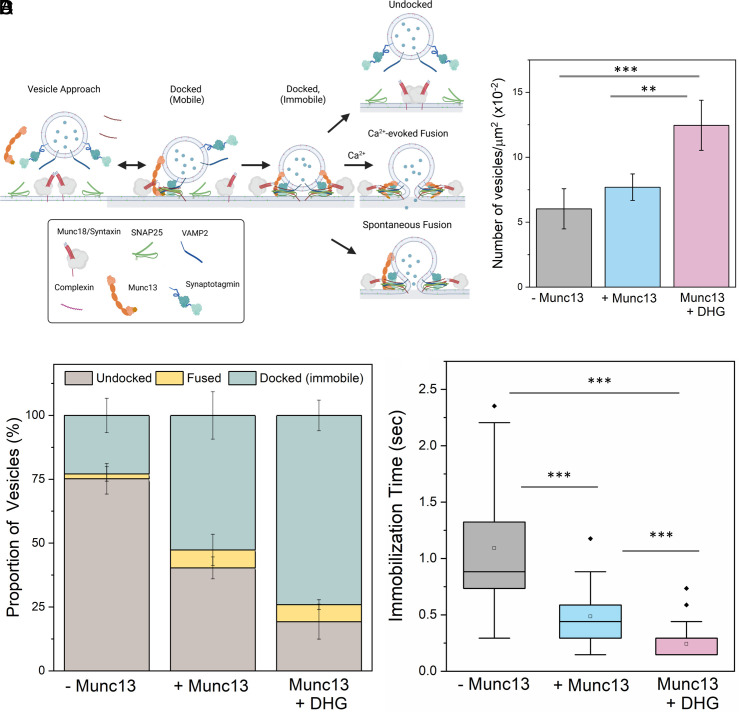
Munc13 and DHG promote the formation of stably, docked “immobile” vesicles. (*A*) To reconstitute the Munc13/Munc18-dependent vesicle priming and fusion process under cell-free conditions, we included a 1:1 Syntaxin/Munc18 complex and palmitoylated SNAP25 in the suspended lipid membrane, as well as VAMP2/Synaptotagmin in SUVs, with CPX and Munc13 in solution. We monitored the fate of a large number of individual vesicles (~200 per condition) using fluorescent labels in the SUVs. (*B*) The number of vesicles attached or “docked” to the suspended bilayer did not appreciably change without or with Munc13 (gray and blue bar, respectively), but DHG activation (pink bar) of Munc13 significantly increased the number of docked vesicles. (*C*) The fate of the docked vesicles strongly depended on the availability of Munc13 and its activation by DHG. In the absence of Munc13 or DHG, most vesicles undock (brown bar), with only a small proportion converting into the immobile docked stage (green bar). The inclusion of Munc13 increased the number of immobile vesicles, which was further enriched by the addition of DHG. In all cases, we observed a small percentage of spontaneous fusion events (yellow bar). (*D*) DHG and Munc13 also accelerated the formation of the immobile docked stage. Without Munc13 or DHG, the docked vesicles reached the immobile state over 1 to 2 s. The addition of Munc13 lowered this transition time to ~0.5 s, which was further reduced by DHG to ~0.25 s. The average values and SDs from three to four independent experiments (with ~200 vesicles per condition) are shown. **P < 0.05; ***P < 0.005 using the Student’s *t* test.

To dissect the molecular mechanisms underlying Munc13-1 function, we adapted our in vitro single-vesicle fusion assay (13, 40, 42) to directly investigate the Munc13-dependent vesicle docking and fusion process (Fig. 1). To best approximate the physiological conditions, we reconstituted Syntaxin with its chaperone Munc18 as a 1:1 functional complex along with palmitoylated SNAP25 at a very low concentration (1:3,200 protein:lipid ratio) in the suspended lipid membrane containing 15% PS and 3% PIP2 (*SI Appendix*, Fig. S1) (43). We employed small unilamellar vesicles (SUVs) with an average of 74 copies (outward facing) of VAMP2 and 25 copies of Synaptotagmin-1 (outward facing) and included Complexin-1 (CPX, 2 µM) in the solution (*SI Appendix*, Fig. S1). In all experiments, we used a conserved C-terminal portion of Munc13-1 consisting of the contiguous C1-C2B-MUN-C2C domains (Munc13-1 residues 529 to 1,735; henceforth referred to as Munc13), except for residues 1,408 to 1,452 within a nonconserved loop in the MUN domain, which were deleted and replaced with EF residues to minimize dimerization and oligomerization (21, 44). We monitored large ensembles of vesicles (~200) and used fluorescently labeled lipid (2% ATTO647N-PE), or a content dye (sulforhodamine B) introduced in the SUVs to track the docking, undocking, clamping, and fusion (either spontaneous or Ca^2+^-evoked) of individual vesicles (*SI Appendix,* Fig. S2).

In the absence of Munc13, most of the docked vesicles remained mobile on the bilayer surface, with the majority of vesicles undocking (~79%) or fusing spontaneously (~2%). Consequently, only a small fraction (~19%) of vesicles converted into an “immobile” (clamped) state and remained unfused during the initial 3-min observation window (Fig. 1 *A*–*C*). Adding Munc13 (200 nM) to the solution resulted in a small (~20%) increase in the total number of docked vesicles (Fig. 1*B*), but it significantly reduced the undocking of vesicles (~40%). The level of spontaneous fusion during this period was low (~8%) resulting in a large fraction (~52%) of the vesicles remaining stably docked, immobile and unfused (Fig. 1 *B* and *C*). The addition of Munc13 also lowered the time the docked vesicles took to reach the immobile “clamped” state from ~1.1 s to ~500 ms (Fig. 1*D*).

**Fig. 2.**
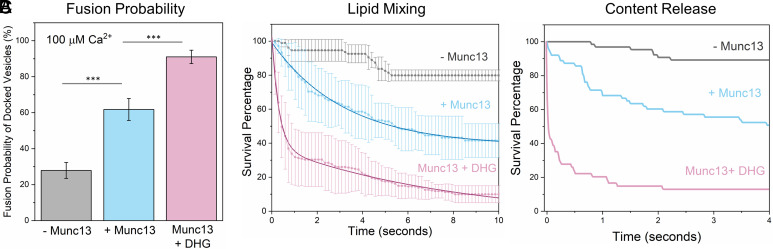
DHG activation of Munc13 is required for rapid, Ca^2+^-evoked vesicular release. The effect of Munc13 and DHG on Ca^2+^ (100 µM)-triggered fusion was evaluated using both lipid mixing (ATTO647N) and content-release (sulforhodamine-B) assay. Calcium Green, a Ca^2+^-sensor dye, introduced in the suspended bilayer (via a lipophilic 24-carbon alkyl chain) was used to monitor the arrival of Ca^2+^ at or near the docked vesicles. (*A*) End-point analysis (from lipid mixing assay) at 1 min post-Ca^2+^-addition revealed that in the absence of Munc13 or DHG (grey bar), only ~25% of stably clamped immobile vesicles fused following Ca^2+^ addition as compared to ~60% when Munc13 was included (blue bar). Activation of Munc13 by DHG (pink bar) further enhanced the fusion probability with ~90% of docked, immobile vesicle fusing in response to the Ca^2+^ signal. Kinetic analyses using lipid mixing (*B*), or content release (*C*) show that Munc13 and DHG also enhance the kinetics of Ca^2+^-evoked fusion. To provide a measure of Ca^2+^-evoked fusion kinetics, we monitored large ensembles of immobile docked vesicles to determine the percent remaining unfused (“survival analysis”) as a function of time elapsed after Ca^2+^-addition and present the results as a “survival curve.” The vesicles clamped in the presence of Munc13 and DHG fused rapidly following Ca^2+^-addition (at t = 0), with the majority fusing <1 s, compared to slower fusion kinetics observed in the absence of DHG and/or Munc13. In the absence of DHG, the average survival trace was best fitted using a one-phase decay nonlinear regression analysis (solid blue line) which yielded a half-life (t_1/2_) ~2.2 s. In the presence of DHG, the survival traces were best approximated using a two-phase decay nonlinear regression analysis (solid red line), which yielded a fast t_1/2_ ~200 ms and a slow t_1/2_ ~5.7 s. These data suggest that DHG activation of Munc13 is essential for ultra-fast Ca^2+^-regulated vesicle fusion. (*A* and *B*) The average values and SDs from three independent experiments (with ~200 vesicles per condition) are shown. **P < 0.05, ***P < 0.005 using the Student’s *t* test. Note: The fusion kinetics of ~50 sulforhodamine-B-loaded vesicles per condition are shown in (*C*). The small number of vesicles precluded detailed statistical analysis. The nonlinear regression was performed using the built-in nonlinear regression functions with least squares regression in Prism (GraphPad Software Inc.).

The addition of Ca^2+^ (100 µM) triggered the fusion of the stably, clamped vesicles (Fig. 2) but to varying degrees depending on prior conditions. In the absence of Munc13, Ca^2+^ influx triggered fusion (as measured by lipid mixing at 147 ms frame rate) of ~25% of docked immobile vesicles, while the inclusion of Munc13 increased the proportion of Ca^2+^-evoked fusion to ~60% of the docked vesicles (Fig. 2*A*). Munc13 also accelerated the fusion kinetics, with the majority fusing within 6 s after Ca^2+^-addition with an estimated half-life (t_1/2_) of ~2.2 s (Fig. 2*B*). In contrast, without Munc13, the vesicles fused slowly and intermittently over 10 s (Fig. 2*B*). In these experiments, we used a lipid-conjugated Ca^2+^ indicator (Calcium green C24) attached to the planar bilayer to estimate the time of arrival of Ca^2+^ at/near each docked vesicle (42). Overall, our data showed that the inclusion of Munc13 significantly improved the formation of stably docked vesicles, along with the probability and kinetics of Ca^2+^-evoked fusion. However, the extent of priming and efficiency of subsequent release for this Munc13-dependent reaction was much lower than when the requirement for Munc13 and Munc18 chaperones was by-passed by employing preassembled t-SNAREs, where >90% of vesicles remained stably docked and fused synchronously with a fusion rate <100 ms (13, 39, 42). This suggested that additional components might be crucial for optimal Munc13 function.

### DAG Activation of Munc13 Is Required to Achieve Fast, Ca^2+^-Coupled Vesicle Fusion.

Since DAG and phorbol esters are known to activate Munc13-1 by binding to its C1 domain (32, 37), we investigated the effect of DAG on Munc13-mediated vesicle docking and fusion. The addition of 1% DAG to the lipid bilayer increased the number of docked vesicles, but most of them (~90%) fused spontaneously (*SI Appendix*, Fig. S3). This significantly reduced the number of docked immobile vesicles, thus hindering the study of Ca^2+^-evoked fusion characteristics. We observed similar behavior with as little as 0.1% DAG in the bilayer (*SI Appendix*, Fig. S3). We have observed that DAG forms separate domains within the bilayer on which Munc13 clusters, irrespective of the bulk concentration (45–47). This limits the ability to effectively vary the local concentration of DAG.

To obviate these limitations, we utilized a short-chain, water-soluble DAG 1,2-hexanoyl-sn-glycerol (DHG), which allowed us to rigorously control both its concentration and the order of addition. To determine the optimal DHG concentration, we incubated the bilayer with various concentrations of DHG (ranging from 0.25 to 1 μM) for 5 min before adding Munc13 (along with SUVs and CPX) and then evaluated its effect on vesicle docking and fusion (*SI Appendix*, Fig. S4). We observed minimal effects of DHG on vesicle docking and fusion at concentrations below 500 nM, while higher concentrations (750 nM or 1 μM) increased the probability of spontaneous fusion events without significantly altering the overall fusion probability, essentially recapitulating the results with long-chain DAG (*SI Appendix*, Fig. S4). We chose to use 500 nM of DHG (which correlates to an effective DAG concentration of ~0.7 mol%) in our experiments for detailed analysis.

The addition of 500 nM DHG to activate Munc13 increased the number of docked vesicles by ~40% (Fig. 1*B*). Notably, most of these vesicles (~75%) remained stably docked in an immobile clamped state (Fig. 1*C*) and reached this state in about 250-ms postdocking (Fig. 1*D*). Upon the addition of Ca^2+^ (100 µM), ~90% of these docked vesicles fused within 8 s (Fig. 2). Within this envelope, there were two distinct populations of vesicles. Approximately 70% fused with a half-life (t_1/2_) of 200 ms, while the remaining ~30% fused (lipid mixing) much more slowly (t_1/2_ ~5.7 s) (Fig. 2*B*). We also tested and confirmed these findings with a content-release assay using a smaller set of sulforhodamine B-loaded SUVs under similar experimental conditions (Fig. 2*C* and *SI Appendix*, Fig. S5) but with a faster video frame rate (13 ms). Control experiments without Munc13 showed DHG alone does not change the vesicle docking, priming and fusion characteristics (*SI Appendix*, Fig. S6). Overall, our findings indicate that Munc13 activation by DAG significantly enhances the formation of stably docked RRP-like vesicles, and is essential for achieving efficient and fast, Ca^2+^-evoked fusion of these docked vesicles.

### Munc13 Promotes and Accelerates the SNARE Complex Assembly.

**Fig. 3.**
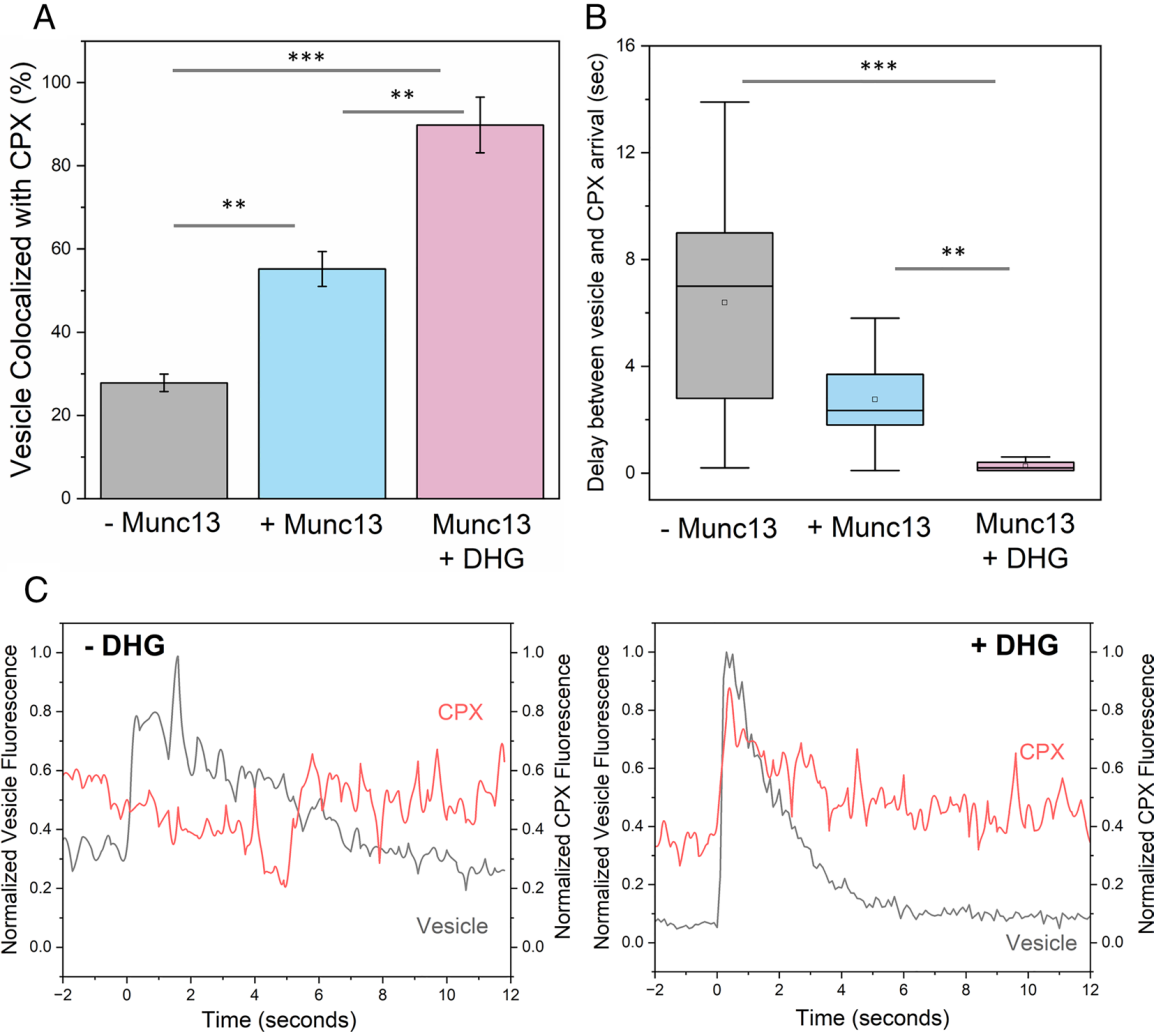
Munc13 and DHG promote and accelerates SNARE complex formation under docked vesicles. The formation of SNAREpins under the docked vesicles (labeled with ATTO465) was visualized by the binding of Alexa Fluor 647-labeled Complexin (CPX). (*A*) Colocalization analysis revealed that in the absence of Munc13 and DHG (gray bar), only about 25% of vesicles contained CPX signals. The proportion of vesicles colocalized with CPX improved to ~55% when Munc13 was included (blue bar) and to ~90% with DHG activation. This indicated that Munc13 directly promotes the SNARE complex formation, and its chaperone function is further stimulated by DHG binding. (*B*) Munc13 and DHG also significantly reduced the delay between vesicle docking and CPX binding, with CPX arrival within ~0.3 s with DHG activation, compared to a 2 to 3 s delay with Munc13 alone and an 8 to 10-s delay without Munc13 or DHG. (*C*) Representative fluorescence traces of vesicle (black) docking and CPX (red) arrival in the presence of Munc13 without or with DHG are shown. (*A* and *B*) The average values and SDs from three independent experiments (with ~100 vesicles per condition) are shown. **P < 0.05, ***P < 0.005 using the Student’s *t* test.

We then investigated whether Munc13’s ability to enhance vesicle docking and fusion is related to its SNARE chaperone function. We utilized the binding of fluorescently labeled CPX, which only binds to preassembled SNAREpins with a 1:1 stoichiometry (10, 42, 48), under TIRF conditions to monitor the efficiency and speed of SNARE complex formation under docked vesicles for different reconstitution conditions (Fig. 3).

In the absence of Munc13 or DHG, approximately 25% of docked vesicles colocalized with CPX. We also observed significant and variable delays between vesicle docking and the arrival of CPX molecules, with an average delay of around 6 s (Fig. 3). When Munc13 was present, the delay was reduced to about 3 s, and around 55% of vesicles colocalized with CPX. DHG addition greatly improved the efficiency of CPX binding (now ~90% of docked vesicles contained CPX) and greatly reduced the delay in CPX binding (delay of approximately 300 ms). As a control, we tested and verified that CPX mutations (R48A Y52A K69A Y70A; CPX^4A^) that disrupt its interaction with SNAREpins completely abolished its colocalization with the docked vesicles (*SI Appendix,* Fig. S7). Our data demonstrate that Munc13 promotes and accelerates the formation of SNAREpins under release-ready vesicles and the Munc13-chaperoned SNAREpin assembly is dramatically accelerated (>10-fold) by DAG binding. Notably, the CPX colocalization levels observed under different reconstitution conditions (Fig. 3) are correlated with the fusion probability (Fig. 2), suggesting that the efficiency of SNAREpin formation, i.e., SNARE priming could account for the observed fusion characteristics.

### Validation of the Munc13/DHG-Dependent Reconstitution.

Previous in vitro reconstitutions (16, 17, 49) have shown DAG to stimulate Munc13-dependent Ca^2+^-triggered vesicle fusion, but its necessity was not established. Therefore, it was important to establish the physiological relevance of our DHG-dependent system. To this end, we used the optimized reconstitution protocol (with 500 nM DHG) and a comprehensive suite of mutations whose mechanism of action with respect to Munc13 activation of SNARE assembly has been established (26, 30, 44, 50). This included Munc13 mutations that disrupt Syntaxin activation (N1128A/F1131A; Munc13^NFAA^) and VAMP2 recruitment (D1358K; Munc13^DK^) (21, 24, 30); as well as a SNAP25 variant (SNAP25^G4S^), in which the last 25 residues (residue 113 to 138) in the linker region were replaced with a flexible, nonspecific GGGGS repeat sequence, rendering it unable to bind to Munc13 (*SI Appendix*, Fig. S8).

**Fig. 4.**
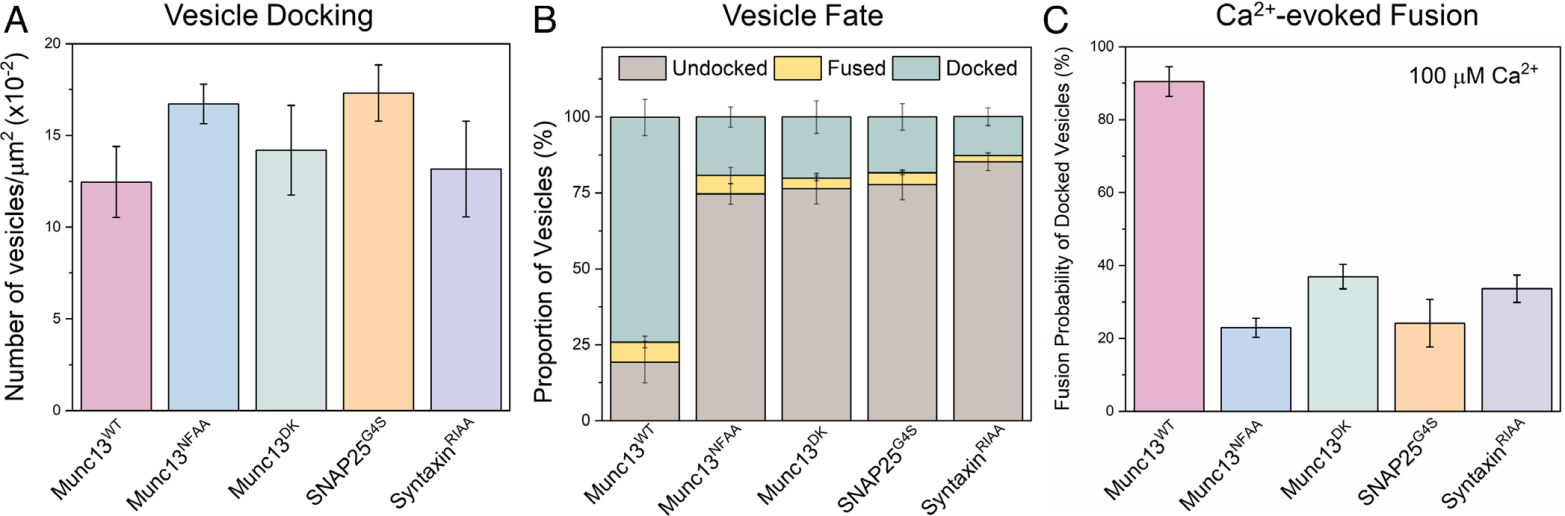
Munc13 interaction with all three SNARE proteins is required for its chaperone function. We employed targeted mutations in Munc13 and SNARE proteins to investigate the functional significance of the Munc13–SNARE interaction in DHG-activated conditions. We used established mutations in Munc13 that disrupt its interaction with Syntaxin (Munc13^NFAA^, blue) or VAMP2 (Munc13^DK^, green) and a reciprocal Syntaxin variant (Syntaxin^RIAA^, purple). Additionally, we designed and tested an SNAP25 linker mutant (SNAP25^G4S^, orange) that is incapable of binding Munc13 (*SI Appendix*, Fig. S6). (*A*) The total number of docked vesicles was similar across all conditions, indicating that the mutations did not affect the initial attachment of vesicles, i.e., docking. (*B*) Disrupting Munc13's interaction with any of the SNARE proteins caused most of the vesicles to ultimately dissociate from the bilayer (undocked). (*C*) The remaining few immobile (“docked”) vesicles failed to fuse upon Ca^2+^ (100 μM) addition. The average values and SDs from three independent experiments (with ~200 vesicles per condition) are shown.

None of the SNARE-binding mutants affected the total number of vesicles that attach during the observation (3 min) period (Fig. 4*A*). However, now a vast majority of initially docked vesicles eventually undock (~75%) or fuse spontaneously (~5%) during the initial observation period, with only a minor fraction (~20%) remaining stably docked (Fig. 4*B*). A further 20% of these immobile, “clamped” vesicles fused upon adding 100 µM Ca^2+^, representing only ~5% of the originally docked vesicles. Similar behavior was observed (Fig. 4) with a complementary Syntaxin mutation (R151A/I155A, Syntaxin^RIAA^) that abrogates its interaction with Munc13 (30). These mutations resulted in outcomes indistinguishable from omitting the Munc13 protein altogether (Figs. 1 and 2) implying that the same binding sites on Munc13 required for neurotransmitter release in vivo and SNARE assembly in vitro are also required for the priming of release-ready vesicles in the fully defined reconstituted system.

### Munc18 and Munc13 Cooperative to Facilitate Priming of Reconstituted Release-Ready Vesicles.

**Fig. 5.**
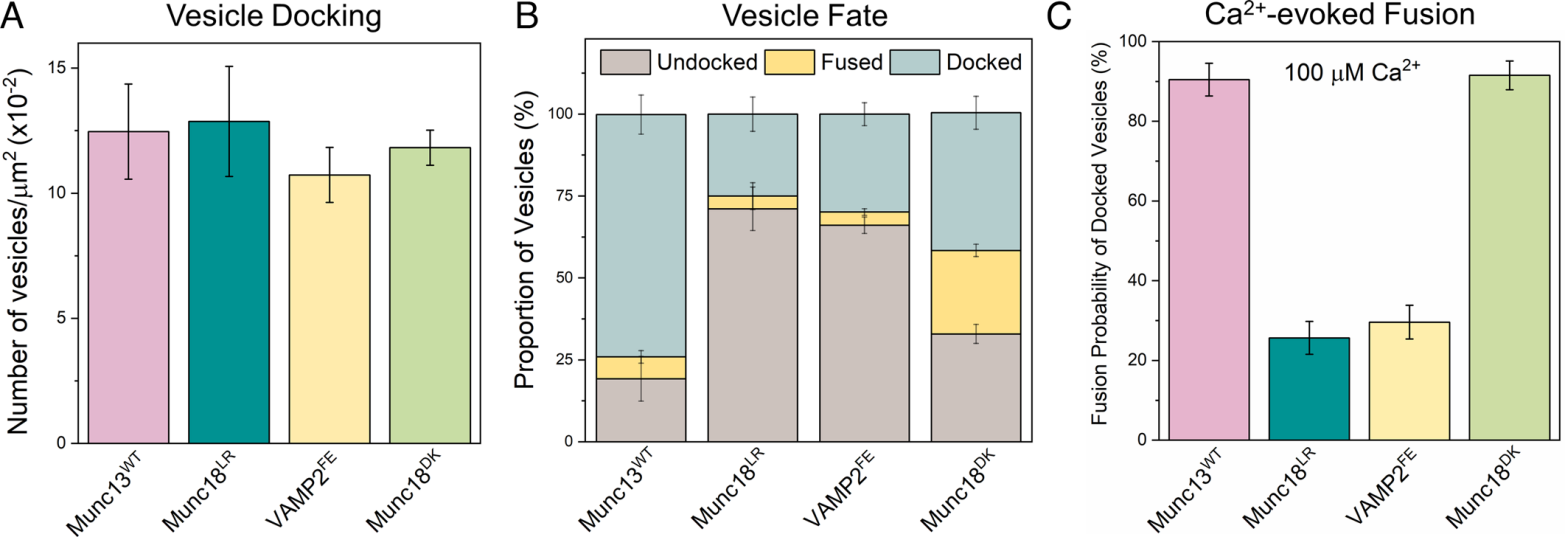
Munc18 and Munc13 act cooperatively to facilitate SNARE complex assembly via the “template” complex. To investigate the role of the Munc18-Syntaxin-VAMP2 “template” complex in the Munc13-mediated vesicle priming and fusion process, we examined the effects of mutations that either disrupt (Munc18^LR^, teal; VAMP2^FE^, yellow) or promote (Munc18^DK^, green) template complex formation. (*A*) We observed a similar number of vesicles for all mutations tested, suggesting that template complex formation is not essential for vesicle docking. (*B*) The fate of the docked vesicles, however, differed depending on the mutation. Negative mutations (Munc18^LR^ and VAMP2^FE^) led to vesicle undocking, while the positive mutation (Munc18^DK^) potentiated spontaneous fusion. Overall, both the positive and negative mutations decreased the number of docked immobile vesicles. (*C*) Disrupting template complex formation also abolished the Ca^2+^-evoked fusion of docked vesicles. Taken together, the data indicate that template complex formation is necessary for productive SNARE complex formation and the coordinated action of Munc13 and Munc18 is required to generate stably docked vesicles. The average values and SDs from three independent experiments (with ~200 vesicles per condition) are shown.

We also assessed the importance of the Munc18-Syntaxin-VAMP2 template complex (23, 25, 51) in the Munc13/DHG-regulated vesicle priming and fusion process (Fig. 5). We found that mutations in Munc18 (L348R, Munc18^LR^) or VAMP2 (F77E, VAMP2^FE^), which destabilize the template complex (26, 51), significantly reduced the number of immobile docked vesicles (from ~75 to ~25%) and the probability of Ca^2+^-evoked fusion (from ~85 to ~20%) of the docked vesicles (Fig. 5). Complementary mutations in Munc18 (D326K, Munc18^DK^) that promote or stabilize the template complex (26, 50) also impaired the formation of the immobile docked state but did not affect their fusogenicity (Fig. 5). In particular, the Munc18^DK^ reduced undocking (from ~70 to ~33%) but strongly increased spontaneous fusion (increasing from ~5 to ~25%). As a result, the proportion of immobile docked vesicles was reduced to ~40%, but the vast majority of these docked vesicles (~85%) still fused upon Ca^2+^ addition indicating that they had sufficient SNARE assembly. Notably, the omission of Munc13 starkly changed the probability and kinetics of Ca^2+^-evoked fusion involving the Munc18^DK^ mutant (*SI Appendix*, Fig. S9). In the presence of Munc13, Ca^2+^-coupled release occurred with >90% probability and within 2 s. In the absence of Munc13, only 50% of docked vesicles fused, and it extended >10 s post-Ca^2+^ addition (*SI Appendix*, Fig. S9). As expected, none of the mutants tested affected the number of docked vesicles (Fig. 5). Taken together, our data indicate that the Munc18-Syntaxin-VAMP2 template complex is a key functional intermediate in the SNARE assembly pathway and is required for the formation of release-ready vesicles and for their subsequent Ca^2+^-evoked fusion in the fully reconstituted system and coordinated action of both Munc13 and Munc18 is required.

## Discussion

Here, we introduce a biochemically defined fusion system that provides the most physiologically accurate in vitro reconstitution of synaptic fusion machinery to date. The system includes the reconstitution of the functional Syntaxin:Munc18 complex along with palmitoylated SNAP25 into a pore-spanning lipid bilayer mimicking presynaptic membranes. This represents a significant improvement over previous efforts, which involved either preassembled t-SNAREs (bypassing the need for both Munc18 and Munc13) or soluble Munc18 and SNAP25 added in large excess (13, 17, 39, 42, 49). These advances were facilitated by the development of optimized protocols enabling us to routinely purify a stable 1:1 Syntaxin:Munc18 complex using Triton X-100 detergent and in vitro palmitoylation of SNAP25 (see *Materials and Methods* for details). Combining this bilayer system with SUVs mimicking synaptic vesicles containing physiologically relevant numbers of VAMP2 and Synaptotagmin allowed us to dissect the interplay between Munc18, Munc13, and DAG in vesicle docking, priming, and Ca^2+^-evoked fusion in molecular terms.

We find that DHG acts as an essential cofactor and is required for optimal Munc13 chaperone function (Figs. 1 and 2). The activation of Munc13 by DAG significantly improved the formation of stably docked RRP-like vesicles and is essential for achieving high-efficiency Ca^2+^-evoked release. However, the effect of short-chain DAG was concentration-dependent—it facilitated Ca^2+^-evoked response at low concentrations but potentiated both spontaneous and evoked fusion at higher concentrations. The behavior observed under high DHG concentration closely resembles the effect of phorbol ester on neurotransmitter release in cultured neurons (52).

We observed that the inclusion of long-chain DAG (even at 0.1%) in the bilayer resulted in extensive spontaneous vesicle fusion, so we used DHG, a soluble DAG analogue, in our experiments. DHG has the same headgroup as DAG and thus, is expected to bind to the Munc13 C1 domain similarly with comparable affinity. Indeed, adding DHG in solution (500 nM) promoted Munc13 binding to lipid bilayers and to a similar extent as including 1% long-chain DAG in the bilayer (*SI Appendix*, Fig. S10*A*). DHG, similar to DAG, promoted the formation of Munc13 clusters on the membrane surface (*SI Appendix*, Fig. S10*B*) further arguing that DHG is a suitable substitute for DAG. We suspect that the formation of DAG-rich domains, which are likely to separate from the fluid phospholipid bilayers (45–47), accounts for the discrepancy between DAG and DHG in our experimental condition.

This phenomenon is likely created by what amounts to an in vitro artifact because such DAG islands are unlikely to exist in neuronal active zones where the abundance of DAG is dynamically and spatially limited and occurs in the context of PIP_2_-rich nanodomains (53, 54). DAG is produced locally by the hydrolysis of PIP_2_ by Ca^2+^-regulated phospholipase C, and the excess DAG is rapidly converted to phosphatidic acid by diacylglycerol kinase and ultimately recycled to PIP_2_ (54, 55). The coordinated action of these enzymes likely ensures that DAG is presented to Munc13, and other active zone proteins as a molecularly dilute and dispersed component mixed within the ~70 nm diameter PIP_2_-rich nanodomains that are known to assemble cluster around Syntaxin both in vivo and in reconstituted bilayers (56). Dynamic alterations of DAG levels in response to Ca^2+^ signals have been shown to play a role in short-term plasticity. Specifically, the activation of the Munc13 C_1_ domain by DAG resulting from Ca^2+^-dependent activation of phospholipase C has been shown to potentiate Munc13 function, resulting in short-term synaptic facilitation (38, 57). The precise mechanism by which DAG activates Munc13 function remains uncertain. DAG binding could potentiate Munc13 by increasing its membrane association and/or cluster formation (*SI Appendix,* Fig. S10). Notably, Munc13 clustering has been demonstrated to promote both vesicle capture and recruitment of SNAP25. Moreover, the C1 domain, in conjunction with the C2B domain, has been shown to play an inhibitory role and DAG binding is predicted to alleviate this inhibition and enhance Munc13 activity (38). Additional structure–function analysis of the C1 domain/DAG interaction is needed to resolve the relative contribution of different molecular mechanisms.

By using fluorescent CPX binding as a label for SNAREpin formation (Fig. 3), we confirmed that the ability of Munc13 to stimulate vesicle fusion probability and kinetics is directly linked to its SNARE chaperone function. Specifically, we find that the improved fusion characteristics observed with Munc13 ± DAG are directly correlated with the enhanced efficacy of SNARE complex formation. This suggested that the SNARE priming process is the rate-limiting step in the vesicle fusion pathway. Supporting this proposition, we have observed a large proportion of stably docked immobile vesicles with very little to no undocking when copurified t-SNARE complex is used (13, 39, 42), as it is expected to enhance the rate of productive ternary SNARE complex formation.

Systematic mutational analyses confirmed that Munc13 and Munc18 act cooperatively, with intersecting interactions with individual SNARE proteins, to guide efficient SNARE complex assembly. Munc18 binds to Syntaxin and VAMP2 to create an intermediate Munc13-Syntaxin-VAMP2 template complex that is critical for productive SNARE complex formation and vesicle fusion. Munc13's chaperone function involves interactions with all three individual SNARE proteins. It accelerates and stabilizes the template complex by promoting proper alignment of Syntaxin and VAMP2. Additionally, Munc13 recruits SNAP25 to the “template” complex, stimulating ternary SNARE complex formation.

Our data further suggest that the activities of Munc18 and Munc13 are closely coordinated and must be balanced for effective clamping of SNAREpins to produce stably docked vesicles and to elicit fast Ca^2+^-evoked vesicular fusion. This implies that SNARE assembly occurs in stages, with sequential and overlapping interactions with chaperones (Munc18 and Munc13) and regulatory elements (Synaptotagmin and Complexin). This ensures that a specific number of SNAREpins are produced and then firmly held in a release-ready state, to facilitate cooperative zippering and ultrafast release upon Ca^2+^ influx. How this occurs in molecular terms is not known.

Insight into the possible mechanism can be gleaned from recent cryoEM structural analyses which revealed that Munc13 undergoes distinct structural transitions involving different oligomeric states (27). We posit that these topological transitions, which are implied by the structures observed to be intimately linked to DAG and Ca^2+^-binding, likely choreograph the SV docking/priming process (27). As such, Munc13 functions as a molecular scaffold that helps to organize the exocytic proteins into a functional complex that is necessary for efficient vesicular release. The in vitro fusion system we describe here provides a unique, focused, and powerful tool to test this specific and other molecular models, and progress toward the goal of deciphering the pointillistic details of SNARE priming by Munc18 and Munc13.

## Materials and Methods

### Materials.

The following cDNA constructs previously described (13, 39, 44) were used in this study: full-length VAMP2 (mouse His^6^-SUMO-VAMP2-, residues 1-116); full-length SNAP25 (mouse His^6^-SNAP25b, residues 1 to 206); Synaptotagmin (rat Synaptotagmin1-His^5^, residues 57 to 421); Complexin (human His^6^-Complexin 1, residues 1 to 134) and Munc13 C1-C2B-MUN-C2C domain (rat His^12^-Munc13-1, residues 529 to 1,735 with residues 1,408 to 1,452 replaced by the sequence EF and 1,532 to 1,550 deleted). We generated an expression clone in the pET28vector to express and purify the Munc18/Syntaxin complex (rat His^6^-SUMO Munc18/Syntaxin1). Phusion High Fidelity Mastermix (New England Biolabs, Ipswich, MA) was used to generate variants in Munc13 (Munc13^NFAA, DK^) VAMP2 (VAMP2^FE^), Syntaxin (Syntaxin^RIAA^) and Munc18 (Munc18^LR, DK^). The SNAP25 linker mutants (SNAP25^G4S^) were synthesized (Genewiz, Inc., Plainfield, NJ) with the SNAP25 loop residues 82 to 105, 97 to 126, and 113 to 138 replaced with (GGGGS) repeats and cloned into the pET28 vector similar to the SNAP25^WT^ construct.

Lipids including 1, 2-Dioleoyl-sn-glycero-3-phosphocholine (DOPC), 1, 2-Dioleoyl-sn-glycero-3-phospho-L-serine (DOPS), L-α-phosphatidylinositol-4, 5-bisphosphate (Brain PIP2) and 1, 2-Dioctadecanoyl-sn-glycerol (DAG) were purchased from Avanti Polar Lipids. Fluorescent lipids ATTO465-DOPE and ATTO647N-DOPE were purchased from ATTO Tec. 1, 2-Dihexanoyl-sn-glycerol (DHG) was purchased from Cayman Chemicals. Alexa Fluor 568/647 – Maleimide and Tris (2-carboxyethyl) phosphine hydrochloride (TCEP) were purchased from Thermo Fisher. Calcium Green conjugated to a lipophilic, 24-carbon alkyl chain (Calcium Green C24) was custom synthesized by Marker Gene Technologies.

### Protein Expression and Purification.

All SNARE and associated proteins were expressed and purified as described previously (13, 39, 44). Briefly, the proteins were expressed in Escherichia coli strain BL21(DE3) (Novagen) and cells were lysed with a cell disruptor (Avestin) in lysis buffer containing 50 mm HEPES, pH 7.4, 400 mM KCl, 2% Triton-X 100, 10% glycerol, 1 mm TCEP, and 1 mm phenylmethylsulfonyl fluoride. Samples were clarified using a 45 Ti rotor (Beckman Coulter) at 140,000 × *g* for 30 min and incubated with Ni-NTA agarose (Thermo Fisher) for 4 to 16 h at 4 °C. The resin was subsequently washed (three column volumes) in the wash buffer (50 mM HEPES, 400 mM KCl, 10% Glycerol, 1 mM TCEP, 32 mm imidazole) with no detergent (Complexin and SNAP25) or 1% Octylglucoside (VAMP2 and Synaptotagmin) or 1% Triton X-100 (Munc18/Syntaxin). Note: We carried out systematic detergent (Octylglucoside, Sodium Cholate, CHAPS, Dodecyl Phosphocholine, Triton X-100) screening to identify 1% Triton X-100 as the ideal detergent to produce stable and functional Munc18/Syntaxin complex. The proteins were either eluted with 300 mM Imidazole (Synaptotagmin) or cleaved off the resin with either Thrombin (SNAP25 and Complexin) or SUMO protease (VAMP2 and Munc18/Syntaxin) in HEPES buffer for 2 h at room temperature. SNAP25 and Complexin proteins were further purified using gel filtration (Superdex75 Hi-load column, GE Healthcare), and Synaptotagmin-1 protein was subjected to anion exchange chromatography (MonoS, GE Healthcare) to remove nucleotide contaminants. The peak fractions were pooled and concentrated using filters of appropriate cutoffs (EMD Millipore). SNAP25 was then palmitoylated using a 20-fold excess of Palmitoyl Coenzyme A (Sigma Aldrich) in HEPES buffer supplemented with 1% TritonX-100 for 30 min at room temperature with gentle mixing. Native mass spectrometry analysis confirmed the palmitoylation of SNAP25 molecule (*SI Appendix*, Fig. S11*A*) and combined with float-up analysis, we estimate 30 to 50% of proteins are palmitoylated.

Munc13 was expressed in ExpiHEK-293 cell cultures using ExpiFectamine as a transfection reagent (Thermo Fisher). Briefly, thawed cells were passaged three times prior to transfection and were grown for 72 h before being spun down and rinsed in ice-cold PBS. The pellet was resuspended in lysis buffer and lysed using a Dounce homogenizer. The sample was clarified at 140,000 *g* for 30 min at 4 °C and the supernatant was incubated overnight with Ni-NTA beads, in the presence of DNAse1, RNAseA, and Benzonase to remove nucleotide contamination. The protein was further washed in the lysis buffer (without Triton-X 100) before being cleaved with PreScission protease for 2 h at room temperature. The eluted proteins were further purified via gel filtration (Superdex 200, GE Healthcare Chicago).

In all cases, the protein concentration was determined using a Bradford Assay (Bio-Rad, Hercules), with bovine serum albumin (BSA) as a standard, and protein purity was verified using SDS/PAGE analysis with Coomassie stain. All proteins were flash-frozen and stored at −80 °C for long-term storage.

### Suspended Bilayer and Vesicle Preparation.

For the suspended bilayer, we approximated the presynaptic membrane physiological composition with 81% DOPC, 15% DOPS, 3% PIP2, and 1% ATTO465-PE for visualization. The lipids were mixed and dried under N_2_ gas followed by a vacuum. For a subset of experiments, we included Ca^2+^-sensor, calcium-green containing a 24-carbon alkyl chain during the sample preparation. Bilayer samples were rehydrated with Munc18/Syntaxin1 and palmitoylated SNAP25 (1:3,200 protein: lipid input ratio) in 5× buffer (125 mM HEPES, 600 mM KCl, 1 mM TCEP, pH 7.4) supplemented with 2% TritonX-100 for 30 min. Samples were then mixed directly with SM-2 Biobeads (Bio-Rad) for 30 min with gentle agitation. The samples were further dialyzed overnight in 5× buffer without detergent. We used mass spectrometry to verify the complete removal of Triton X-100 (*SI Appendix*, Fig. S11*B*). We used a liposome float-up assay to verify that Munc18/Syntaxin remains a stable 1:1 complex on the membrane even in the presence of pSNAP25 (*SI Appendix*, Fig. S1*B*). Indeed, we find approximately 1:1:1 ratio of Syntaxin:Munc18:pSNAP25 following reconstitution and float-up (*SI Appendix*, Fig. S1*B*). Furthermore, we observed that unpalmitoylated SNAP25 (added in solution) does not float up along with Munc18/Syntaxin further suggesting that we do not form appreciable amount of Syntaxin/SNAP25 complex. This indicates that our reconstitution reflects the physiological starting point of these proteins in presynaptic membranes.

Lipid bilayers were created by drying and rehydrating membranes to form GUVs as previously described (40). Briefly, 4 µL drops of Munc18/Syntaxin/SNAP25 containing proteoliposomes were dried on a clean Mattek dish and rehydrated twice. In the second rehydration, the sample was diluted 5× to 20 µL with distilled water and then added to a cleaned silicon chip containing 1× buffer (25 mM HEPES, 120 mM KCl, 1 mM TCEP, pH 7.4) supplemented with Mg^2+^ (5 mM). The bilayer was extensively washed with 1× buffer, and the fluidity of the suspended bilayer was verified using fluorescence recovery after photobleaching using ATTO465 (lipid) or Alexa568 (Protein) fluorescence labels. Consistent with a fluid and mobile bilayer, the average diffusion coefficient of the lipid was calculated to be 3.8 µm^2^/s while the protein diffusion coefficient was found to be 1.7 µm^2^/s (*SI Appendix*, Fig. S12). For TIRF experiments, we created the suspended bilayer using the redesigned silicon chip (41), with buffer supplemented with 45% OptiPrep gradient media for index matching (41). Optiprep on the top was washed out with 1× buffer after bilayer formation. Bilayers contained 0.1% ATTO465 and were photobleached with 100% laser power after verifying they had formed.

For small-unilamellar vesicle preparation, we approximated the synaptic vesicle lipid composition using 83% DOPC, 15% DOPS, and 2% ATTO647N-PE. The samples were dried under N_2_ gas followed by a vacuum. Lipids were rehydrated with VAMP2 (1:100) and Synaptotagmin1 (1:250) in buffer (140 mM KCl, 50 mM HEPES, 1 mM TCEP, pH 7.4) supplemented with 1% Octyl β-glucoside. After 30 min of mixing, samples were rapidly diluted 3× below CMC and allowed to sit for another 30 min before being dialyzed overnight buffer without detergent. The samples were subjected to additional purification on the discontinuous Nycodenz gradient. For content-release assays, we prepared vesicles supplemented with 25 mol% cholesterol in the lipid formulation with 30 mM sulforhodamine-B. Samples were subjected to gel filtration using a CL4B column to remove the unincorporated dye and were further purified as described above. Based on the densitometry analysis of coomassie-stained SDS gels and chymotrypsin digest, we estimated vesicles contained ~74 and ~25 copies of outward-facing VAMP2 and Syt1 respectively.

### Single-Vesicle Fusion Assays.

Single-vesicle assays were performed as described previously (13, 42) with a few modifications. DHG (at the defined concentration) was added to the preformed bilayer and incubated for 5 min. Vesicles (500 nM lipids) were added from the top using a pipette and allowed to interact with the bilayer for 3 min. We used the ATTO647-PE or sulforhodamine-B fluorescence to track the fate of the individual vesicles. All vesicles that attach to the suspended bilayer during the 3-min observation period were considered as “docked” vesicles. Initially, all docked vesicles exhibited diffusive mobility and subsequently transitioned to an “immobile” clamped state (*SI Appendix,* Fig. S2). Among these mobile and immobile vesicles, some underwent spontaneous fusion, evident by a burst of fluorescence intensity followed by a rapid decrease. Furthermore, we observed instances of undocking, where vesicle fluorescence disappeared without a fusion burst, primarily during the mobile phase (*SI Appendix,* Fig. S2). Undocking events were infrequent once vesicles reached the immobile state.

After the initial 3-min interaction phase, the excess vesicles in the chamber were removed by buffer exchange (3× buffer wash) and 100 µM CaCl_2_ was added from the top to monitor the effect of Ca^2+^ on the docked vesicles. The lipid mixing experiments were recorded at a 147-ms frame rate to track large numbers (~30 to 40 vesicles) simultaneously. For the content-release assay, the frame rate was increased to 13 ms by adjusting to a smaller region of interest and thus, tracking fewer vesicles at a time. As before, the increase in calcium green fluorescence at 532 nm was used to precisely estimate the arrival of Ca^2+^ at or near the docked vesicle (*SI Appendix*, Fig. S4).

### Complexin-Binding Assay.

To track the binding of Complexin to docked vesicles, we used the current iteration of the silicon chips, which are compatible with TIRF microscopy (41). For these colocalization experiments, vesicles were labeled with 2% ATTO465-PE and the sole cysteine residue in Complexin1 was labeled with AlexaFluor 647-Maleimide, with >85% efficiency. The bilayer was photobleached prior to the addition of vesicles, mixed with labeled CPX and Munc13, without or with preincubation with DHG. We used a DuoView 2 system for simultaneous tracking of vesicles and CPX in the 488 and 647 channels. All experiments were carried out at a 100-ms frame rate on the TIRF (Nikon) microscope.

### Microscale Thermophoresis Interaction Analysis.

Microscale thermophoresis (MST) analysis was carried out as described previously (44). Briefly, Halo-tagged Munc13-1 construct was labeled with AlexaFluor 660, and MST analysis was carried out with titration of SNAP25 (1 nM to 125 µM range) into 50 nM of fluorescently labeled Munc13-1 constructs in a premium-coated glass capillary using a Monolith NT.115 (Nanotemper Technologies).

### Cryo-Electron Microscopy Analysis.

The cryo-electron microscopy analysis of Munc13 binding to lipid membrane was carried out described previously (27). Preformed vesicles (DOPC/DOPS/PIP2 in a molar ratio of 14/80/6) were diluted down to lipid concentration of 100 µM and mixed with 0.25 μM Munc13C protein in 1:1 (vol/vol) ratio. Once mixed, the samples were incubated at room temperature for 5 min then kept on ice until freezing. Samples (2.5 μL) were vitrified using a Vitrobot Mark IV (Thermo Fisher Scientific) held at 8 °C with 100% humidity. They were applied to freshly glow-discharged 200-mesh Lacey Formvar/carbon grids, and grids were blotted for 5 s with blot force −1 and then plunged frozen in liquid ethane cooled by liquid nitrogen. Samples were imaged using Glacios Cryo TEM 200 kV (Thermo Fisher Scientific) equipped with a K2 Summit direct electron detector (Gatan).

### Munc13 Clustering Assay.

We adapted the protocol described previously (36, 44) to assess the formation of Munc13 clusters on lipid membrane surface in the absence or presence of DAG/DHG. Liposomes were prepared with following lipid composition (71% DOPC, 25% DOPS, 2% PIP2, ±2% DAG) using extrusion method with HEPES buffer (50 mM HEPES, 140 mM KCl, 1 mM TCEP, pH 7.4). Lipid bilayers were created by Mg^2+^ (5 mM) induced bursting liposomes in ibidi glass-bottom chambers (ibidi GmbH, Germany) and extensively washed with the HEPES buffer supplemented with EDTA (6 mM). Munc13-Halo-Alexa488 (10 nM) was added to the prewashed bilayer and incubated for 60 mins. The samples were imaged on a TIRF (Nikon) microscope with a 63× oil objective. For DHG experiments, we added 500 nM DHG is solution along with Munc13.

### Mass Spectrometry Analysis.

To carry out mass spectrometry analysis, all samples were buffer exchanged to 200 mM ammonium acetate with Zeba™ spin desalting columns (Thermo Fisher Scientific), with an approximate protein concentration of 2 to 10 µM. Stable electrospray ionization was achieved using in-house nanoemitter capillaries in Q Exactive UHMR (Thermo Fisher Scientific). The nanoemitter capillaries were formed by pulling borosilicate glass capillaries (O.D–1.2 mm, I.D–0.69 mm, length–10 cm, Sutter Instruments) using a Flaming/Brown micropipette puller (Model P-1000, Sutter Instruments). After the tips were formed using this puller, the nanoemitters were coated with gold using rotary pumped coater Q150R Plus (Quorum Technologies). To perform the mass spectrometry–based measurement, the nanoemitter capillary was filled with buffer-exchanged protein samples and installed into Nanospray Felx™ ion source (Thermo Fisher Scientific). The MS parameters were optimized as per the samples of analysis. The typical parameters were as follows: spray voltage was in the range of 1.2 to 1.5 kV, capillary temperature was 275 °C, the resolving power of the MS was between 3,125 and 6,250 at m/z of 400, the ultrahigh vacuum pressure of 5.51e^−10^ to 6.68e^−10^ mbar, the in-source trapping range between −50 V and −200 V. The HCD voltage was optimized for each sample to achieve a good-quality spectra, and the range was between 0 and 200 V. All the mass spectra were visualized and analyzed with the Xcalibur software.

## Supplementary Material

Appendix 01 (PDF)

## Data Availability

All study data are included in the article and/or *SI Appendix*.
